# An empirical comparison of methods for analyzing correlated data from a discrete choice survey to elicit patient preference for colorectal cancer screening

**DOI:** 10.1186/1471-2288-12-15

**Published:** 2012-02-20

**Authors:** Ji Cheng, Eleanor Pullenayegum, Deborah A Marshall, John K Marshall, Lehana Thabane

**Affiliations:** 1 Department of Clinical Epidemiology and Biostatistics, McMaster University, Hamilton, ON, Canada; 2 Biostatistics Unit, St. Joseph's Healthcare Hamilton, Hamilton, ON, Canada; 3 Department of Community Health Sciences, University of Calgary, Calgary, AB, Canada; 4 Department of Medicine, Division of Gastroenterology, McMaster University, Hamilton, ON, Canada; 5 Biostatistics Unit/FSORC, 3rd Floor Martha, Room H325, St. Joseph's Healthcare Hamilton, 50 Charlton Avenue East, Hamilton, ON L8N 4A6, Canada

**Keywords:** Discrete choice experiment, Intra-class correlation, Statistical model, Patient preference

## Abstract

**Background:**

A discrete choice experiment (DCE) is a preference survey which asks participants to make a choice among product portfolios comparing the key product characteristics by performing several choice tasks. Analyzing DCE data needs to account for within-participant correlation because choices from the same participant are likely to be similar. In this study, we empirically compared some commonly-used statistical methods for analyzing DCE data while accounting for within-participant correlation based on a survey of patient preference for colorectal cancer (CRC) screening tests conducted in Hamilton, Ontario, Canada in 2002.

**Methods:**

A two-stage DCE design was used to investigate the impact of six attributes on participants' preferences for CRC screening test and willingness to undertake the test. We compared six models for clustered binary outcomes (logistic and probit regressions using cluster-robust standard error (SE), random-effects and generalized estimating equation approaches) and three models for clustered nominal outcomes (multinomial logistic and probit regressions with cluster-robust SE and random-effects multinomial logistic model). We also fitted a bivariate probit model with cluster-robust SE treating the choices from two stages as two correlated binary outcomes. The rank of relative importance between attributes and the estimates of ***β ***coefficient within attributes were used to assess the model robustness.

**Results:**

In total 468 participants with each completing 10 choices were analyzed. Similar results were reported for the rank of relative importance and ***β ***coefficients across models for stage-one data on evaluating participants' preferences for the test. The six attributes ranked from high to low as follows: cost, specificity, process, sensitivity, preparation and pain. However, the results differed across models for stage-two data on evaluating participants' willingness to undertake the tests. Little within-patient correlation (ICC ≈ 0) was found in stage-one data, but substantial within-patient correlation existed (ICC = 0.659) in stage-two data.

**Conclusions:**

When small clustering effect presented in DCE data, results remained robust across statistical models. However, results varied when larger clustering effect presented. Therefore, it is important to assess the robustness of the estimates via sensitivity analysis using different models for analyzing clustered data from DCE studies.

## Background

With increased emphasis on the role of patients in healthcare decision making, discrete choice experimental (DCE) designs are more often used to elicit patient preferences among proposed health services programs [1,2]. DCE is an attribute-based design drawn from Lancaster's economic theory of consumer behaviour [3] and the statistical principles of the design of experiments [4]. This method measures consumer preference according to McFadden's random utility (benefit) maximisation (RUM) framework amongst a choice set which contains two or more alternatives of products or goods varying along several characteristics (attributes) of interest. In the early 1980s, Louviere, Hensher and Woodworth [5,6] introduced DCE into marketing research, and since then DCE has been rapidly adopted by researchers in other areas such as transportation, environment and social science. Its applications in health research emerged in the early 1990s, and it has been increasingly used to evaluate patient preferences for currently available and newly-proposed health services or programs in health economics and policy-making related topics. For example, in the health economics related research area, 34 published studies used DCE design in the period from 1990 to 2000, and 114 DCE design studies were published in the period from 2001 to 2008 [7].

In the short history of using DCE in health research, there were several reviews [7-9], and debates about methodological and design issues, challenges and future development [10-12]. In generating a DCE study, three major formats of the choice design have frequently been used: i) a forced choice between two alternatives, ii) a choice among three or more alternatives with an opt-out option, and iii) a two-staged choice process which forces participants to choose one of the alternatives and then an opt-out choice is provided to allow participants to say no to all proposed products [13]. Despite the rapid developments in design aspects [12,14], less attention was paid to the statistical analysis and model selection issues. Lancaster and Louviere [15] and Ryan and et al. [13] discussed several statistical models used for DCE including multinomial logistic model (MNL), multinomial probit model (MNP), and mixed logit model (MIXL). However, these studies did not provide detailed comparisons amongst competing models, or a clear indication of how to best deal with model selection issues. Another aspect related to the analysis of DCE data is adjustment for clustering effects. For example, in the DCE survey, it is common to ask participants to respond to several choice tasks in one survey. Each choice task has the same format but different attribute combinations. Naturally the choices made by same person would be expected to be more similar than the choices of other persons, leading to the within-patient correlation of responses. This within-subject correlation caused by the clustering effects or repeated observations needs to be accounted for in the analysis [16]. It is often measured using the intra-class correlation coefficient (ICC) where ICC = 0 indicates no intra-person correlation and ICC = 1 indicates perfect intra-person correlation. In this paper, we empirically compared some commonly-used statistical models which also account for the clustering effects in DCE analysis. We assessed the robustness (consistency and discrepancy) of the models on ranking of the relative importance between the attributes and the estimates of the ***β ***coefficients within each level of the attributes.

The data we used were taken from the preference survey on colorectal cancer (CRC) screening tests conducted in Hamilton, Ontario, Canada in 2002 [17]. This project used a two-level choice design. Thus, the data structure allowed us to investigate the statistical models for analyzing binary, nominal and bivariate outcomes for DCE data.

## Methods

### Overview of the CRC screening project

The Canadian Cancer Society reported in 2011 that CRC is the fourth most commonly diagnosed cancer and the second leading cause of cancer death in Canada [18]. According to the same report, the estimates of new cases of CRC and CRC related death in 2011 were 22,200 (50 per 100,000 person) and 8,900 (20 per 100,000 persons) in 2011. Although CRC has a high incidence rate, patients have a better chance of successful treatment if diagnosis can be made earlier. Although a population-based CRC screening program is highly recommended for people over 50 years of age [19,20], the uptake rate in North America is only about 50% [21]. Therefore, better understanding of patient preferences for screening tests may be the key to the successful implementation and uptake of CRC screening programs. This survey was the first conducted in Canada to evaluate patient preferences for various CRC screening tests to identify the key attributes and levels that may influence CRC screening test uptake.

Traditional CRC screening modalities such as fecal occult blood testing (FOBT), flexible sigmoidoscopy (SIG), colonoscopy (COL) and double-contrast barium enema (DCBE) vary on their process, accuracy, comfort and cost [22]. In this survey, five important attributes of features of the screening tests were identified through review of the literature, consultation with clinical specialists and patient focus groups. They were: process (4 levels), pain (2 levels), preparation (3 levels), specificity (3 levels) and sensitivity (3 levels). In addition, cost (4 levels) was included due to its potential influence on the uptake (Table 1). To reduce the burden on respondents for making their choices on 864 (4 × 2 × 3 × 3 × 3 × 4) unique combination from full factorial design, we used a fractional factorial design. In this design, 40 choice tasks were divided into four blocks to create a subset of 10 choice tasks of the attribute combinations for each survey participant to evaluate. The original design was developed using the SAS Optex procedure and optimized several measures of efficiency: 1) level balance; 2) orthogonality; and 3) D-efficiency [17,23]. This design ensured the ability of estimating the main effects of the attributes while minimizing the number of combinations. No prior information on the ranking of attributes from the literature was available at the time of the design of the study. The survey used the pair-wise binary two-stage response design [24] with the choice between two choice sets of the attributes at different levels as the first step and the addition of an opt-out option as the second step (Table 2). This design maximized the information gained through the questionnaire to understand patient preferences on the CRC screening tests and the factors affecting the uptake rate. However, the analysis presented challenges. First, the answers were likely to cluster within subjects because each subject made two sequential choices for ten choice tasks. Therefore, a statistical model adjusting for within-subject correlation for repeated measurements was needed. Second, in the original paper, the analysis was done using the bivariate probit model, but the analysis could be approached using different methods: treating the responses at the two stages as independent responses, as sequential and correlated bivariate responses, or as a single response with three levels (Test A, Test B or No screening).

**Table 1:** Attributes and Levels Used in the Stated Preference Survey

Attributes	Attribute description as presented to patients	Levels	Level description as presented to patients
Process	How is it done?	Stool	You place 2 stool samples onto special cards for 3 consecutive days and return them to your doctor
		Scope	A flexible tube with a small camera at the tip is inserted into your rectum and through your colon
		CT	You lie on a special table while a machine moves around you and takes x-ray pictures (like a CAT scan)
		Enema and X-ray	Air and a white liquid are injected into your colon through a rectal tube. x-ray pictures are taken as the liquid moves through your colon*
Pain	Is there pain or discomfort?	None	You feel no pain during the test
		Mild	You may feel mild pain or discomfort during the test*
Preparation	What do you do to prepare?	None	No preparation required
		Diet	You must alter your diet for 5 days by avoiding some specific foods and over-the-counter medications
		Enema/lax	Before the test you must take laxatives or enemas which cause diarrhea to clean your colon*
Specificity	Is it accurate if you DO NOT have cancer?	100%	If you DO NOT have cancer, the test result will never say you may have cancer. No other test is needed.
		80%	If you DO NOT have cancer, the test result will say you may have cancer 2 out of 10 times. You then need to have a different test done
		50%	If you DO NOT have cancer, the test result will say you may have cancer 5 out of 10 times. You then need to have a different test done.*
Sensitivity	Is it accurate if you DO	90%	If you DO have cancer, the test will miss it 1 out of 10 times
		70%	If you DO have cancer, the test will miss it 3 out of 10 times
		40%	If you DO have cancer, the test will miss it 6 out of 10 times*
Cost	How much would you pay?	$10	$10*
		$50	$50
		$250	$250
		$500	$500

*Reference level for attribute

**Table 2:** A sample question

Features	Test A	Test B
How is it done?	You place 2 stool samples onto special cards for 3 consecutive days and return them to your doctor	A flexible tube with a small camera at the tip is inserted into your rectum and through your colon
Is there pain or discomfort?	You feel no pain during the test	You may feel mild pain or discomfort during the test
What do you do to prepare?	You must alter your diet for 5 days by avoiding some specific foods and over-the-counter medications	Before the test you must take laxatives or enemas which cause diarrhea to clean your colon
Is it accurate if you DO NOT have cancer?	If you DO NOT have cancer, the test result will say you may have cancer 5 out of 10 times. You then need to have a different test done	Same as for Test A
Is it accurate if you DO have cancer?	If you DO have cancer, the test will miss it 3 out of 10 times	Same as for Test A
How much would you pay?	$50	$250
Which test would you prefer (please mark one box only)	Prefer A	Prefer B
Suppose you now have the option of no screening. What would you prefer now?(please mark one box only)	I would still prefer the test chosen above	
	I would prefer no screening	

### Outcomes

**Figure 1:**
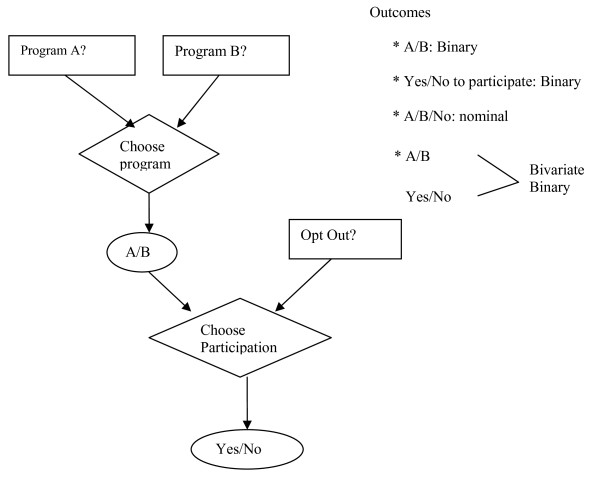
**Two-stage design and outcomes for analysis**.

According to the unique data structure of the two-stage design, we conducted three analytic approaches. 1) Analyze the two-staged sequential choices of each choice task separately, i.e. binary outcomes: a) subject preferences on the screening modalities which only included patient responses at the first stage, and b) subject willingness to participate in the screening program which only included subjects' responses at the second stage. 2) Treat the two-staged data as paralleled three-choice options including Test A, Test B and "opt-out", i.e. nominal data. 3) Treat the two-staged data as two correlated binary choice sets, i.e. bivariate outcomes. Figure 1 presents the data structure of the original design and these three analysis approaches.

### Random utility theory

As mentioned above, the DCE design is generally based on random utility theory [25] which expresses the utility (benefit) *U_in _*of an alternative *i *in a choice set *C_n _*(perceived by individual *n*) as two parts: 1) an explainable component specified as a function of the attributes of the alternatives *V(X_in_, β)*; and 2) an unexplainable component (random variation) *ε_in_*.

$$U_{in}=V\left(X_{in},\beta \right)+\varepsilon_{in}$$

The individual *n *will choose alternative *i *over other alternatives if and only if this alternative gives the maximized utility. The relationship of the utility function and the observed *k *attributes of the alternatives can be assumed under a linear-in-parameter function.

$$V_{in}=\alpha_{\text{i}}+\beta_{\text{i}}{x_{\text{i}}}_{1}+\ldots +\beta_{\text{k}}x_{\text{ik}}$$

According to the assumption of the distribution of the error term *ε_in_*, the models specification of DCE data can be varied.

### Statistical methods

The statistical models discussed in this paper were organized according to the type of outcomes: i) logistic and probit models for binary outcomes, ii) multinomial logistic and probit models for nominal outcomes, and iii) bivariate probit model for bivariate binary outcomes. We provide some details on how the different statistical techniques account for the within-cluster correlation in analyzing clustered DCE data.

For the binary type of outcomes, we examined six statistical models which have the capacity to account for the within-patients correlations [26,27], including logistic regression with clustered robust standard error, random-effects logistic regression, logistic model using generalized estimating equations (GEE), probit regression with clustered robust standard error, random-effects probit regression, and probit regression using generalized estimating equation (GEE) model. Below are some brief descriptions of the methods.

#### Standard logistic regression and standard probit regression

Both standard logistic and probit regressions assume that the observations are independent. However in our dataset, each subject completed ten choice tasks, i.e. each subject had ten observations (choice tasks) which formed a cluster or can be considered repeated measurement. Normally, the observations in the same clusters are more similar (correlated) comparing to the observations out of the cluster. Therefore, adjusting the correlation within the cluster is necessary. We used three methods to adjust the within-cluster correlation.

#### Clustered robust standard error

In this method the independence assumptions are relaxed among all observations, but it is assumed that the observations across clusters are independent. The total variance is empirically estimated using Huber-White (also called Sandwich) standard error [28]. This method takes only the intra-class correlation into account, but the degrees of freedom are still based on the number of observations, not the number of clusters [29]. Therefore, this method only adjusts the standard error related to the confidence interval, but the point estimates are left unchanged.

#### Random-effects method

In this method, the total variance has two components: between-cluster variance and within-cluster variance. We assume that, at the cluster level, data follow a normal distribution with mean zero and between-cluster variance τ^2^; and that within each cluster, data vary according to some within-cluster variance [30]. This method takes two types of variance into account when estimating the total variance and the degrees of freedom are calculated based on the number of clusters [31]. Therefore, the point estimates and their corresponding variances are adjusted for intra-cluster correlation. For the covariance structure, we assumed equal variances for the random effects and a common pairwise covariance [32]. This structure corresponds to the exchangeable correlation structure specified for GEE method, which we describe below. The key difference between the random-effects method and other methods discussed here is that the random-effects method estimates the parameters for each subject within cluster or clusters sharing the same random effects. Therefore, the random effect is also often called subject specific effect [33].

### GEE method

This method allows a working correlation matrix to be specified to adjust the within-cluster correlation. We assumed that there was no ordering effect among the observation in each cluster, allowing us to use an exchangeable correlation matrix [34]. As in the random-effects method, the degrees of freedom are based on the number of clusters, which in turn adjusts the estimate of the confidence interval [35]. Unlike the random-effects method, the GEE approach estimates the regression parameters averaging over the clusters (so-called population average model) [36].

For the nominal type of outcomes, we used three statistical models [37]: multinomial logistic model with clustered robust standard error, random-effects multinomial logistic model, and multinomial probit model with clustered robust standard error. We also fitted a bivariate probit model in which the choices from two stages were treated as two binary outcomes [38].

#### Multinomial logistic model

McFadden's conditional logit model (CLM), also called multinomial logistic (MNL) model, was the pioneer and most commonly used model in the early DCE studies [39]. The key assumption of this model is that the error terms *ε_in _*are independent and identically distributed (IID) [13], which leads to the independence of irrelevant alternatives (IIA) property [40]. Another assumption for this model is that the error term has an extreme value distribution with mean 0 and variance π^2^/6 [37]. To take the intra-class correlation into account, the clustered robust SE was used.

#### Random-effects multinomial logistic model

Similar to the random-effects models used for analyzing binary outcomes, this model takes two levels of variance, between-cluster variance and within-cluster variance, into account for clustered or longitudinal nominal responses [41,42].

#### Multinomial probit model

Multinomial probit model (MNP) (heteroscedastic models) is considered to be one of the most robust, flexible and general models in DCE, especially when the correlation (heteroscedasticity) between alternatives is presented [43]. The model is assumed to have a normally distributed error term. The benefit of using MNP model is that the IIA assumption which is the strict requirement for MNL model can be somehow relaxed [37]. The main concern in using this model is that its maximization involves Monte Carlo simulation but not the analytical maximization which could lead to a computational burden. Again, the clustered robust SE was used to incorporate the intra-class correlation.

#### Bivariate probit model

In this model, we assume that the choices between two stages (stage 1: choice between screening test; stage 2: choice between participation and opt-out) are not independent. It says that subject choice as to whether or not to participate in the screening program was conditional on subject preference for the screening modalities [44]. By fitting this model, two types of correlation can be taken into account: the correlation between the outcomes from stage 1 and stage 2, incorporated through the bivariate nature of the model itself, and the intra-class correlation, incorporated through use of the cluster robust SE.

To assess the necessity of accounting for the intra-class correlation for analyzing clustered correlated DCE data, we also presented the results from the above models using simple standard error (SE)--which does not take clustering into account. They are the standard logistic, probit, multinomial logistic, multinomial probit and bivariate probit models.

We compared results from the above models on the following criteria: rank on the relative importance of the attributes, and magnitude, direction and significance of the estimates of the ***β ***coefficient within each level of the attributes, which were obtained by regressing preference onto the difference in attributes between the two choices. The ranking criterion was measured by the percent change between the log-likelihood value of the full model and the value after removing one specific attribute from the model [45]. To evaluate the significance of the estimate of the ***β ***coefficients within each attribute, the criterion for statistical significance was set at alpha = 0.05. All statistical models were conducted using STATA 10.2 (College Station TX) and the figures were plotted using PASW Statistics 19 (SPSS: An IBM Company).

## Results

**Figure 2:**
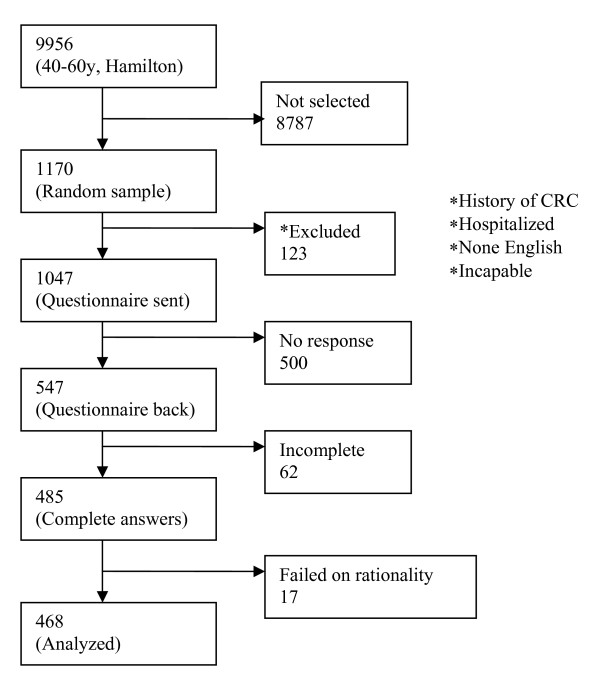
**Flow chart of sample selection**.

A random sample of 1,170 patients was selected from a roster of 9,959 patients aged 40-60 years from the Hamilton Primary Care Network. After excluding the patients who did not pass the inclusion criteria, questionnaires were mailed to 1,049 patients. Of these, 547 were returned and 485 had complete data. Among the patients with complete data, we excluded 17 patients who did not pass the rationale test, which were two warm-up choice tasks. For these warm-up tasks, one alternative was dominant over another possessing all favourable attribute levels and the respondents who did not choose the dominant alternative were considered to have failed the rational test. Finally, we analyzed the data for 468 patients (Figure 2) from four blocks with the block size of 105, 124, 120 and 119 respectively.

The mean age of the subjects was 50.8 years (standard deviation, 5.95 years), which was similar to the recommended age to start CRC screening [46]. Of the 468 included subjects, about 48% were female, 12% had family history of CRC and two patients (0.2%) had been diagnosed with CRC. The detailed demographic characteristics are presented in Table 3.

**Table 3:** Demographic characteristic of respondents

Personal Characteristics	(n = 468)
Age in years: Mean (SD)	50.8 (5.95)
Gender	
Male	52%
Female	48%
Health Status	
Excellent	14%
Very good	42%
Good	33%
Fair	9%
Poor	2%
Family history of CRC	
Yes	12%
No	82%
I don't know	6%
Diagnosed with CRC	
Yes	0.4% (2 patients)
No	99%
I don't know	0.6%

**Figure 3:**
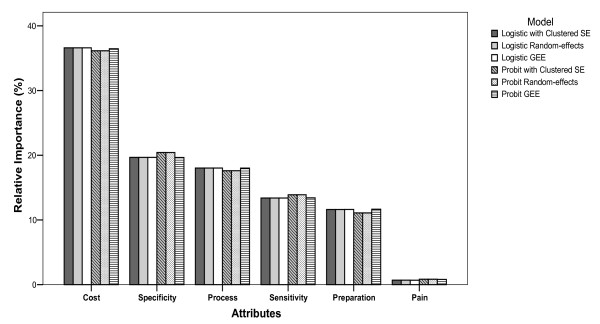
**Relative importance of choice between Test A and Test B (stage-one)**.

**Figure 4:**
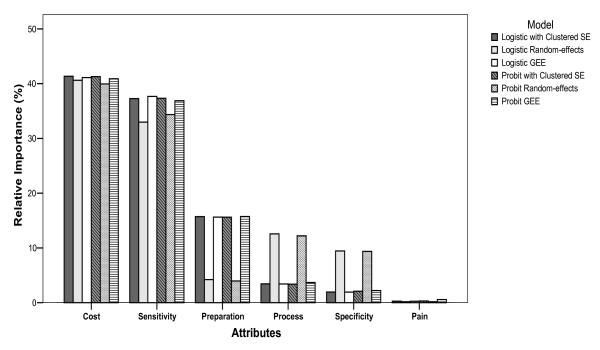
**Relative importance of choice between participation and opt-out (stage-two)**.

**Figure 5:**
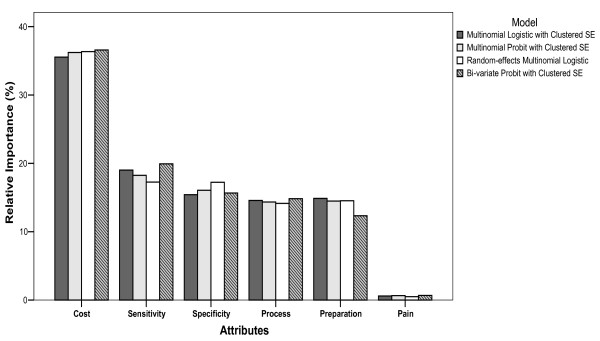
**Relative importance of choice between Test A, Test B and opt-out (combined stage one and two)**.

For the two-point outcomes (binary), the rank of the attributes on the choice of Test A and Test B was consistent across models. From most important to least important, they ranked as follows: cost, specificity, process, sensitivity, preparation and pain (Figure 3). With the exception of the random-effects logistic and probit models, the ranking (from most important to least important) of the six attributes for assessing participation or opt-out (stage-two), was as follows: cost, sensitivity, preparation, process, specificity and pain. The ranking from random-effects models was: cost, sensitivity, process, specificity, preparation and pain (Figure 4). For the three-point outcomes (nominal and bivariate) in which the choices of Test A, Test B and opt-out were estimated simultaneously, the attributes were ranked consistently: cost, sensitivity, specificity, process, preparation and pain (Figure 5). Comparing to the models using simple SE, using clustered robust SE to incorporate intra-class correlation did have any effects on calculating the relative importance of attributes.

When looking at how certain levels of each attribute affected the choice between Test A and Test B (stage-one), the estimates of the ***β ***coefficients were similar in magnitude and direction across different statistical models. The most preferred screening test had the following features: stool sample, no preparation, 100% specificity, 70% sensitivity, without pain and with an associated cost of $50. The least preferred screening test had the combination of colonoscopy, special diet for preparation, 80% specificity, 90% sensitivity, with mild pain and no associated cost (Table 4 and Table 5).

**Table 4:** Estimates of coefficients of patient choice between Test A and Test B (Two-point outcome from stage-one)

				Two-point outcome				
		**Logistic Model**				**Probit Model**		

	**Simple SE**	**Robust SE**	**Random-effects**	**GEE**	**Simple SE**	**Robust SE**	**Random-effects**	**GEE**

Process ref = Enema/X-ray)								
								
Stool	0.27 (0.16, 0.37)	0.27 (0.19, 0.35)	0.27 (0.16, 0.37)	0.27 (0.16, 0.37)	0.16 (0.10, 0.23)	0.16 (0.12, 0.21)	0.16 (0.10, 0.23)	0.16 (0.10, 0.23)
Scope	-0.20(-0.31,-0.09)	-0.20(-0.27,-0.13)	-0.20(-0.31,-0.09)	-0.20(-0.31,-0.09)	-0.12(-0.19,-0.05)	-0.12(-0.17,-0.08)	-0.12(-0.19,-0.05)	-0.12(-0.19,-0.05)
CT	-0.13(-0.24,-0.03)	-0.13(-0.23,-0.04)	-0.13(-0.24,-0.03)	-0.13(-0.24,-0.03)	-0.08(-0.15,-0.02)	-0.08(-0.14,-0.02)	-0.08(-0.15,-0.02)	-0.08(-0.15,-0.02)
No Pain (ref = mild pain)	0.04(-0.03, 0.10)	0.04(-0.01, 0.08)	0.04(-0.03, 0.10)	0.04(-0.03, 0.10)	0.02(-0.01, 0.06)	0.02(-0.001, 0.05)	0.02(-0.01, 0.06)	0.02(-0.01, 0.06)
Preparation (ref = Enema/Lax)								
None	0.17 (0.09, 0.25)	0.17 (0.11, 0.23)	0.17 (0.09, 0.25)	0.17 (0.09, 0.25)	0.10 (0.05, 0.16)	0.10 (0.07, 0.14)	0.10 (0.05, 0.16)	0.10 (0.05, 0.16)
Special Diet	-0.17(-0.26,-0.09)	-0.17(-0.24,-0.11)	-0.17(-0.26, -0.09)	-0.17 (-0.26, -0.09)	-0.11 (-0.16, -0.05)	-0.11 (-0.14, -0.07)	-0.11 (-0.16, -0.05)	-0.11 (-0.16, -0.05)
Specificity (ref = 50%)								
100%	0.14 (0.05, 0.22)	0.14 (0.06, 0.21)	0.14 (0.05, 0.22)	0.14 (0.05, 0.22)	0.09 (0.03, 0.14)	0.09 (0.04, 0.13)	0.09 (0.03, 0.14)	0.09 (0.03, 0.14)
80%	-0.26 (-0.35, -0.18)	-0.26 (-0.33, -0.20)	-0.26 (-0.35, -0.18)	-0.26 (-0.35, -0.18)	-0.17 (-0.22, -0.11)	-0.17 (-0.21, -0.12)	-0.17 (-0.22, -0.11)	-0.17 (-0.22, -0.11)
Sensitivity (ref = 40%)								
90%	-0.07 (-0.15, 0.01)	-0.07 (-0.14, 0.01)	-0.07 (-0.15, 0.01)	-0.07 (-0.15, 0.01)	-0.04 (-0.09, 0.01)	-0.04 (-0.08, 0.01)	-0.04 (-0.09, 0.01)	-0.04 (-0.09, 0.01)
70%	0.24 (0.15, 0.33)	0.24 (0.15, 0.33)	0.24 (0.15, 0.33)	0.24 (0.15, 0.33)	0.15 (0.03, 0.14)	0.15 (0.10, 0.20)	0.15 (0.03, 0.14)	0.15 (0.03, 0.14)
Cost (ref = $10)								
$50	0.63 (0.47, 0.79)	0.63 (0.0.52, 0.74)	0.63 (0.47, 0.79)	0.63 (0.47, 0.79)	0.39 (0.29, 0.49)	0.39 (0.32, 0.46)	0.39 (0.29, 0.49)	0.39 (0.29, 0.49)
$250	0.17 (0.01, 0.33)	0.17 (0.07, 0.28)	0.17 (0.01, 0.33)	0.17 (0.01, 0.33)	0.10 (0.01, 0.20)	0.10 (0.04, 0.17)	0.10 (0.01, 0.20)	0.10 (0.01, 0.20)
$500	0.44 (0.24, 0.63)	0.44 (0.30, 0.58)	0.44 (0.24, 0.63)	0.44 (0.24, 0.63)	0.27 (0.15, 0.39)	0.27 (0.19, 0.36)	0.27 (0.15, 0.39)	0.27(0.15, 0.39)

**Table 5:** Estimates of coefficients of patient choice of Test A and Test B (Stage-one from three-point outcome)

			Three-point of outcome				
			**Nominal**			**Bivariate**	

	**MNL Simple SE**	**MNL Robust SE**	**MNL Random-effects**	**MNP Simple SE**	**MNP Robust SE**	**Bivariate probit Simple SE**	**Bivariate probit Robust SE**
Process (ref = Enema/X-ray)							
Stool	0.24 (0.11, 0.36)	0.24 (0.14, 0.33)	0.23 (0.09, 0.36)	0.18 (0.09, 0.28)	0.18 (0.11, 0.26)	0.16 (0.10, 0.23)	0.16 (0.12, 0.21)
Scope	-0.25 (-0.38, -0.13)	-0.25 (-0.34, -0.17)	-0.29 (-0.42, -0.15)	-0.19 (-0.29, -0.09)	-0.19 (-0.26, -0.11)	-0.12 (-0.18, -0.05)	-0.12 (-0.16, -0.08)
CT	-0.14 (-0.27, -0.01)	-0.14 (-0.25, -0.02)	-0.13 (-0.27, -0.01)	-0.10 (-0.20, -0.001)	-0.10 (-0.19, -0.01)	-0.08 (-0.15, -0.02)	-0.08(-0.14, -0.01)
No Pain (ref = mild pain)	0.04 (-0.03, 0.11)	0.04 (-0.01, 0.09)	0.04 (-0.04, 0.12)	0.04 (-0.01, 0.09)	0.03 (-0.01, 0.07)	0.02 (-0.01, 0.06)	0.02 (-0.01, 0.05)
Preparation (ref = Enema/Lax)							
None	0.18 (0.09, 0.28)	0.18 (0.11, 0.26)	0.18 (0.08, 0.28)	0.14 (0.07, 0.22)	0.14 (0.09, 0.20)	0.10 (0.05, 0.16)	0.10 (0.07, 0.14)
Special Diet	-0.21 (-0.32, -0.11)	-0.21 (-0.29, -0.14)	-0.24 (-0.35, -0.13)	-0.16 (-0.25, -0.08)	-0.16 (-0.22, -0.10)	-0.11 (-0.16, -0.05)	-0.11 (-0.14, -0.07)
Specificity (ref = 50%)							
100%	0.14 (0.04, 0.25)	0.14 (0.04, 0.25)	0.15 (0.04, 0.26)	0.11 (0.03, 0.19)	0.11 (0.03, 0.19)	0.08 (0.03, 0.14)	0.08 (0.04, 0.13)
80%	-0.29 (-0.39, -0.19)	-0.29 (-0.37, -0.21)	-0.33 (-0.44, -0.23)	-0.22 (-0.30, -0.14)	-0.22 (-0.29, -0.16)	-0.17 (-0.22, -0.11)	-0.17 (-0.21, -0.12)
Sensitivity (ref = 40%)							
90%	-0.07 (-0.17, 0.03)	-0.07 (-0.14, 0.01)	-0.07 (-0.17, 0.04)	-0.04 (-0.12, 0.03)	-0.04 (-0.10, 0.01)	-0.04 (-0.10, 0.02)	-0.04 (-0.08, 0.01)
70%	0.20 (0.09, 0.31)	0.20 (0.10, 0.30)	0.20 (0.08, 0.32)	0.16 (0.07, 0.25)	0.16 (0.09, 0.24)	0.15 (0.09, 0.21)	0.15 (0.10, 0.20)
Cost (ref=$10)							
$50	0.59 (0.40, 0.78)	0.59 (0.46, 0.72)	0.66 (0.46, 0.85)	0.45 (0.30, 0.60)	0.45 (0.35, 0.55)	0.39 (0.29, 0.49)	0.39 (0.32, 0.46)
$250	0.15 (-0.04, 0.35)	0.15 (0.02, 0.29)	0.14 (-0.06, 0.35)	0.11 (-0.05, 0.26)	0.11 (-0.01, 0.21)	0.10 (0.00, 0.20)	0.10 (0.04, 0.17)
$500	0.52 (0.28, 0.76)	0.52 (0.34, 0.70)	0.57 (0.32, 0.83)	0.39 (0.20, 0.57)	0.39 (0.25, 0.52)	0.27 (0.15, 0.39)	0.27 (0.19, 0.36)

**Figure 6:**
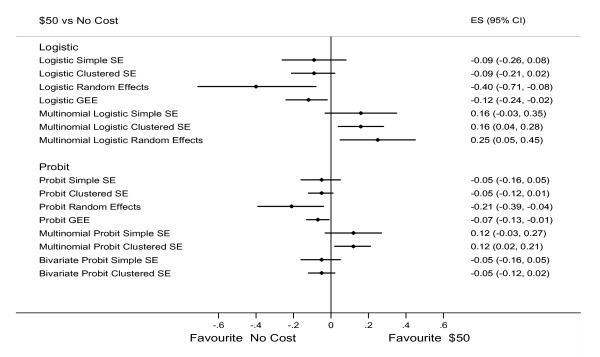
***β *Coefficients with 95% CI (Cost: $50 vs. No cost) of patient choice between participation and opt-out**.

When assessing the impact of certain levels of each attribute on patient choice of participating or opt-out (stage-two), the ***β ***coefficient estimates for 90% sensitivity and no preparation had a significantly positive effect on uptake and this was consistent across all models. For other attributes and levels, results appeared similar across all three global analysis approaches: the random-effects and GEE logistic models and the random-effects and GEE probit models (Table 6); MNL with clustered robust SE, MNL random-effects and MNP with clustered robust SE (Table 7); and logistic with clustered robust SE, probit with clustered robust SE and bivariate probit (Table 6 and Table 7). The following two examples showed the estimates across models could differ by magnitude and direction. The magnitude of estimates of the effect of 90% sensitivity varied by model, but the direction was similar across all models. When comparing the cost of $50 to no cost, logistic and probit random-effects and GEE models reported that participants preferred no cost. MNL with clustered robust SE, MNL random-effects and MNP with clustered robust SE model reported that participants preferred the $50 cost. For other models, no significant statistical differences were found (Figure 6). We also found that unlike the results from the stage-one data (Table 4 and Table 5), for the stage-two data there was noticeable difference between the ***β ***coefficient estimates from the models with and without incorporating the intra-class correlation (Table 6 and Table 7).

**Table 6:** Estimates of coefficients of patient choice of participation or opt-out (Two-point outcome from stage-two)

				Two-point outcome				
		**Logistic Model**				**Probit Model**		

	**Simple SE**	**Robust SE**	**Random-effects**	**GEE**	**Simple SE**	**Robust SE**	**Random-effects**	**GEE**
Process (ref = Enema/X-ray)								
Stool	0.00 (-0.11, 0.11)	0.00 (-0.10, 0.09)	-0.13 (-0.34, 0.08)	-0.04 (-0.10, 0.03)	0.00 (-0.07, 0.07)	0.00 (-0.06, 0.06)	-0.08 (-0.19, 0.04)	-0.02 (-0.06, 0.02)
Scope	-0.02 (-0.14, 0.09)	-0.02 (-0.13, 0.08)	-0.20 (-0.42, 0.01)	-0.06 (-0.13, 0.01)	-0.01 (-0.08, 0.06)	-0.01 (-0.08, 0.05)	-0.10 (-0.22, 0.02)	-0.04 (-0.08, 0.01)
CT	0.08 (-0.03, 0.20)	0.08 (-0.03, 0.20)	0.62 (0.40, 0.85)	0.18 (0.11, 0.25)	0.05 (-0.02, 0.12)	0.05 (-0.02, 0.12)	0.35 (0.22, 0.47)	0.11 (0.07, 0.15)
No Pain (ref = mild pain)	0.01 (-0.05, 0.08)	0.01 (-0.04, 0.07)	0.04 (-0.09, 0.16)	0.01 (-0.03, 0.05)	0.01 (-0.03, 0.05)	0.01 (-0.02, 0.04)	0.02 (-0.05, 0.09)	0.01 (-0.02, 0.03)
Preparation (ref = Enema/Lax)								
None	0.15 (0.06, 0.24)	0.15 (0.07, 0.22)	0.25 (0.09, 0.42)	0.08 (0.03, 0.14)	0.09 (0.03, 0.14)	0.09 (0.04, 0.13)	0.14 (0.04, 0.23)	0.05 (0.02, 0.08)
Special Diet	-0.03 (-0.13, 0.06)	-0.03 (-0.13, 0.07)	-0.01 (-0.20, 0.17)	-0.01 (-0.07, 0.05)	-0.02 (-0.08, 0.04)	-0.02 (-0.07, 0.04)	-0.01 (-0.11, 0.09)	-0.01 (-0.04, 0.03)
Specificity (ref = 50%)								
100%	0.01 (-0.08, 0.10)	0.01 (-0.07, 0.09)	0.34 (0.17, 0.51)	0.10 (0.04, 0.15)	0..01 (-0.05, 0.07)	0.01 (-0.04, 0.06)	0.19 (0.09, 0.29)	0.01 (0.02, 0.09)
80%	0.04 (-0.05, 0.13)	0.04 (-0.03, 0.11)	0.08 (-0.09, 0.25)	0.02 (-0.03, 0.08)	0.02 (-0.03, 0.08)	0.03 (-0.02, 0.07)	0.05 (-0.05, 0.14)	0.01 (-0.02, 0.05)
Sensitivity (ref = 40%)								
90%	0.21 (0.12, 0.30)	0.21 (0.13, 0.29)	0.71 (0.53, 0.89)	0.21 (0.16, 0.27)	0.13 (0.07, 0.18)	0.13 (0.08, 0.18)	0.40 (0.31, 0.50)	0.13 (0.10, 0.16)
70%	0.01 (-0.08, 0.11)	0.01 (-0.07, 0.09)	0.04 (-0.14, 0.22)	0.01 (-0.04, 0.07)	0.01 (-0.05, 0.07)	0.01 (-0.04, 0.06)	0.03 (-0.07, 0.13)	0.01 (-0.03, 0.04)
Cost (ref = $10)								
$50	-0.09 (-0.26, 0.08)	-0.09 (-0.21, 0.02)	-0.40 (-0.71, -0.08)	-0.12 (-0.24, -0.02)	-0.05 (-0.16, 0.05)	-0.05 (-0.12, 0.01)	-0.21 (-0.39, -0.04)	-0.07 (-0.13, -0.01)
$250	-0.16 (-0.36, 0.02)	-0.16 (-0.36, 0.04)	-1.21 (-1.56, -0.86)	-0.36 (-0.47, -0.25)	-0.10 (-0.20, 0.01)	-0.09 (-0.22, 0.02)	-0.67 (-0.87, -0.48)	-0.22 (-0.13, -0.01)
$500	-0.54 (-0.74, -0.34)	-0.54 (-0.74, -0.34)	-1.69 (-2.08, -1.30)	-0.53 (-0.65, -0.41)	-0.33 (-0.45, -0.21)	-0.33 (-0.45, -0.21)	-0.95 (-1.16, -0.73)	-0.32 (-0.40, -0.25)

**Table 7:** Estimates of coefficients of patient choice of participation or opt-out (Stage-two from three-point outcome)

			Three-point of outcome				
			**Nominal**			**Bivariate**	

	**MNL Simple SE**	**MNL Robust SE**	**MNL Random-effects**	**MNP Simple SE**	**MNP Robust SE**	**Bivariate probit Simple SE**	**Bivariate probit Robust SE**
Process (ref = Enema/X-ray)							
Stool	0.09 (-0.03, 0.21)	0.09 (-0.01, 0.19)	0.07 (-0.07, 0.20)	0.08 (-0.01, 0.18)	0.08 (0.01, 0.16)	0.00 (-0.07, 0.07)	0.00 (-0.06, 0.06)
Scope	-0.14 (-0.27, -0.01)	-0.14 (-0.25, -0.03)	-0.23 (-0.37, -0.09)	-0.10 (-0.20, 0.00)	-0.10 (-0.01, -0.19)	-0.02 (-0.08, 0.05)	-0.02 (-0.08, 0.04)
CT	0.04 (-0.09, 0.17)	0.04 (-0.08, 0.15)	0.13 (-0.01, 0.27)	0.02 (-0.08, 0.12)	0.02 (-0.07, 0.11)	0.05 (-0.02, 0.12)	0.05 (-0.02, 0.12)
No Pain (ref = mild pain)	0.03 (-0.04, 0.11)	0.03 (-0.02, 0.09)	0.03 (-0.05, 0.11)	0.03 (-0.03, 0.09)	0.03 (-0.02, 0.07)	0.01 (-0.03, 0.05)	0.01 (-0.02, 0.04)
Preparation (ref = Enema/Lax)							
None	0.23 (0.13, 0.33)	0.23 (0.15, 0.31)	0.20 (0.09, 0.31)	0.17 (0.09, 0.25)	0.17 (0.11, 0.24)	0.09 (0.03, 0.14)	0.09 (0.04, 0.13)
Special Diet	-0.13 (-0.24, -0.02)	-0.13 (-0.24, -0.02)	-0.17 (-0.29, -0.05)	-0.09 (-0.18, -0.01)	-0.09 (-0.17, -0.01)	-0.02 (-0.08, 0.04)	-0.02 (-0.08, 0.04)
Specificity (ref = 50%)							
100%	0.07 (-0.03, 0.17)	0.07 (-0.02, 0.17)	0.16 (0.05, 0.27)	0.06 (-0.02, 0.14)	0.06 (-0.01, 0.13)	0.01 (-0.05, 0.06)	0.01 (-0.04, 0.06)
80%	-0.09 (-0.19, 0.02)	-0.09 (-0.17, -0.01)	-0.17 (-0.28, -0.05)	-0.06 (-0.22, 0.09)	-0.06 (-0.13, -0.01)	0.03 (-0.03, 0.09)	0.03 (-0.02, 0.07)
Sensitivity (ref = 40%)							
90%	0.19 (0.09, 0.29)	0.19 (0.10, 0.27)	0.22 (0.11, 0.33)	0.14 (0.06, 0.22)	0.14 (0.07, 0.0.21)	0.13 (0.08, 0.18)	0.13 (0.08, 0.18)
70%	0.10 (-0.01, 0.21)	0.10 (0.01, 0.20)	0.13 (0.01, 0.25)	0.08 (-0.01, 0.16)	0.08 (0.01, 0.15)	0.01 (-0.05, 0.07)	0.01 (-0.04, 0.06)
Cost (ref=$10)							
$50	0.16 (-0.03, 0.35)	0.16 (0.04, 0.28)	0.25 (0.05, 0.45)	0.12 (-0.03, 0.27)	0.12 (0.02, 0.21)	-0.05 (-0.16, 0.05)	-0.05 (-0.12, 0.02)
$250	-0.08 (-0.28, 0.12)	-0.08 (-0.27, 0.11)	-0.26 (-0.48, -0.04)	-0.07 (-0.22, 0.09)	-0.07 (-0.22, 0.09)	-0.10 (-0.20, 0.01)	-0.10 (-0.22, 0.02)
$500	-0.32 (-0.54, -0.09)	-0.32 (-0.52, -0.11)	-0.28 (-0.63, -0.04)	-0.25 (-0.43, -0.08)	-0.25 (-0.42, -0.09)	-0.33 (-0.45,- 0.21)	-0.33 (-0.45, -0.21)

When assessing the clustering effect, we found that intra-class correlation was small among the stage-one data (ICC ≈ 0) and relatively large among the stage-two data (ICC = 0.659). For this survey, it appears as though many patients had predetermined their participation for CRC screening. For example, among the 468 participants included in the analyses, 48% always chose to undertake the screening program and 15% always chose no participation regardless of how the screening modalities varied at the first stage. Although Test A and Test B were generic terms of the combinations of the different levels of six attributes and they were randomly assigned to appear first or second in one choice task, we found that 24% more participants chose Test A over Test B. All the design limitations had some impact on our interpretation of the analysis results.

## Discussion

We applied six statistical models to binary outcomes, three models to nominal multinomial outcomes and one model to bivariate binary outcomes to estimate the ranking of key attributes of CRC screening tests using data from DCE survey conducted in Hamilton, Ontario, Canada in 2002. We used three methods to adjust the within-cluster correlations: clustered robust standard error, random-effects, and GEE methods. The results showed consistent answers for estimating subject preference for CRC screening tests, both on ranking the importance of the attributes and identifying the significant factors influencing subject choice between testing modalities. For estimating subject willingness to participate or undertake CRC screening (i.e. incorporating "out-put" option), models disagreed both on ranking the importance of the attributes and identifying the significant factors (i.e. attributes and levels) affecting whether or not subjects would participate.

Overall, our analyses showed that participants preferred a CRC screening test with the following characteristics: stool sample, no preparation, 100% specificity, 70% sensitivity and without pain. The CRC test with such a combination of attribute levels would be the FOBT test [18]. Thus, our findings appear to be consistent with the results from Nelson and Schwartz's survey in 2004 [47] which showed FOBT to be the most preferred option for CRC screening. In that survey, they also reviewed 12 previous studies, all of which showed FOBT to be a preferred choice by most patients.

The reason for the consistency in estimating the choice between screening tests and the discrepancy in estimating the choice between participation and "out-put" might be due to the model's ability to adjust the within-participant (cluster) correlation. When the within-cluster correlation is small (choice between Test A and Test B), the assumption of the independently and identically distributed error term *ε_in _*is held. Therefore, it might not be necessary to take the clustering effects into account and thus the estimates are similar across statistical models. However, when the intra-class correlation presents, the analysis needs to account for both the within-cluster variance and between-cluster variance [48].

To the best of our knowledge, this is the first empirical study to compare different methods to address the within-participant correlation in the analysis of DCE data. However, many authors have emphasized the importance of adjusting for clustering in analysis of clustered data or repeated measurements for binary outcomes [49,50]. When intra-class correlations are present in clustered or longitudinal data, the random-effects and GEE models are two commonly recommended approaches. Although they are estimating different parameters (the estimates from random-effects model are interpreted for the observations in the same cluster; the estimates from GEE model are interpreted as the mean across entire sample), the results from these two models are similar most of the time [41,51]. Some researchers generally prefer random-effects model when the results from these two approaches disagree. However, some researchers argue that the random-effects model could provide biased results due to unverifiable assumptions about the data distribution [52].

Comparing to the models for analyzing correlated binary data, statistical software seldom has ready-to-use statistical models developed for multinomial outcomes or multi-variate outcomes. The multinomial probit model is routinely used to deal with correlation between alternatives [53], but it does not take intra-class or intra-respondent correlation into account. Robust standard error can be specified for multinomial logistic or probit and bivariate logistic models to adjust the estimate of standard error, but this would not correct the bias related to point estimates (coefficients). A simulation study has shown that the bias and the inconsistency for estimating the within-cluster correlation increase with the size of the cluster [54]. The newly developed generalized linear latent and mixed model (***gllamm***) procedure in STATA has the ability to run random-effects multinomial logistic model [55] to address the intra-class correlation issue, but this model has yet to be evaluated for performance (i.e. whether or not yields unbiased estimates). Some researchers have suggested using Bayesian hierarchical random-effects logistic and probit regression for clustered or panel data [56]. Although the Bayesian approach allows the flexibility to specify random effects, it requires considerable skill in programming.

This study has some limitations. First, this study is an empirical comparison of the analytic models and therefore we cannot know which model performs the best. Such an analysis would require simulation studies to assess the performance of the models in terms of the bias, precision, and coverage. Second, some estimates of the cost attribute in our study were inexplicable. For the test associated cost, participants' preference had a non linear order: $50, $0, $500 and $250. This could be a result of as the violation of the model assumptions or model misspecification. Most DCE analyses assume a linear utility function, but some recent studies have shown that this assumption may not be true for price-related attributes. A study of MPS players found that the utility function of the price and storage size had W-shaped curves rather than smooth linear trends [57]. A local travel mode study also found that the preference of time savings followed a non-linear utility function [58]. Another reason which may cause inaccurate results in our study is the use of two-staged design. The two-staged design had the advantage of maximizing the information gained by forcing participants to make a choice at the first stage, but it also gave us some artificial information. Third, many respondents in this survey seemed to have predetermined their participation in CRC screening before seeing the questionnaire. This may have caused an unusually high with-in cluster correlation when choosing between participation and opt-out. We also doubt that the predetermination might cause the ordering effect [59] when choosing the preferred screening tests. When individuals are forced to make a choice between products which they have decided that they do not want, the answer might not resemble the truth. Therefore, the results need to be interpreted cautiously--replication from similar studies is needed to better understand participant preferences for CRC screening and the willingness to undertake the screening program.

## Conclusion

Responses from the same participant are likely to be more similar than the responses between participants in DCE data leading to possible intra-class or intra-participant correlation. Therefore, it is important to investigate the size of intra-class correlation before fitting any statistical model. We found that when within-cluster correlation is very small, all models gave consistent results both on the estimates ranking and coefficients. Therefore, the simplest logistic regression and multinomial logistic regression are recommended for the computation advantage being ease. Multinomial probit model may be a preferred choice method of analysis if we assume the existence of the correlation between alternatives.

When within-cluster correlation is high, sensitivity analyses are needed to examine the consistency of the results. Instead of making generalized inferences according to the estimate from any single statistical model, results from the sensitivity analyses based on different models can provide some insight about the robustness of the findings.

Our study empirically compared some commonly used statistical model on taking intra-class correlation into account when analyzing DCE data. To completely understand the necessity of accounting for the intra-class correlation for DCE data, particularly on analyzing nominal type of outcomes, simulation studies are needed.

## Conflict of interest

The authors declare that they have no competing interests.

## Authors' contributions

JC (chengj2@mcmaster.ca) conducted literature review, preformed the statistical analyses and composed the draft of the manuscript. LT (thabanl@mcmaster.ca) designed the original study, oversaw the statistical analysis and revised the manuscript. EP (pullena@mcmaster.ca) assisted planning statistical analyses and revised the manuscript. DAM (damarsha@ucalgary.ca) and JKM (marshllj@mcmaster.ca) designed the original study and revised the manuscript. All authors read and approved the final manuscript.

## Pre-publication history

The pre-publication history for this paper can be accessed here:

http://www.biomedcentral.com/1471-2288/12/15/prepub
